# Tumour necrosis factor‐α‐induced protein 8‐like 2 is a novel regulator of proliferation, migration, and invasion in human rectal adenocarcinoma cells

**DOI:** 10.1111/jcmm.14065

**Published:** 2019-01-13

**Authors:** Dong‐Dong Wu, Shi‐Yu Liu, Ying‐Ran Gao, Dan Lu, Ya Hong, Ya‐Ge Chen, Peng‐Zhen Dong, Da‐Yong Wang, Tao Li, Hui‐Min Li, Zhi‐Guang Ren, Jian‐Cheng Guo, Fei He, Xue‐Qun Ren, Shi‐Yong Sun, Shao‐Feng Duan, Xin‐Ying Ji

**Affiliations:** ^1^ School of Basic Medical Sciences Henan University College of Medicine Kaifeng China; ^2^ Joint National Laboratory for Antibody Drug Engineering Henan International Joint Laboratory for Nuclear Protein Regulation Henan University Kaifeng China; ^3^ The First Affiliated Hospital of Henan University Kaifeng China; ^4^ Center for Precision Medicine Zhengzhou University Zhengzhou China; ^5^ Huaihe Hospital of Henan University Kaifeng China; ^6^ Department of Hematology and Medical Oncology Emory University School of Medicine and Winship Cancer Institute Atlanta Georgia; ^7^ Institute for Innovative Drug Design and Evaluation Henan University School of Pharmacy Kaifeng China

**Keywords:** apoptosis, autophagy, rectal adenocarcinoma, signalling pathway, tumour necrosis factor‐α‐induced protein 8‐like 2

## Abstract

Tumour necrosis factor‐α‐induced protein 8‐like 2 (TIPE2) is a tumour suppressor in many types of cancer. However, the mechanism of action of TIPE2 on the growth of rectal adenocarcinoma is unknown. Our results showed that the expression levels of TIPE2 in human rectal adenocarcinoma tissues were higher than those in adjacent non‐tumour tissues. Overexpression of TIPE2 reduced the proliferation, migration, and invasion of human rectal adenocarcinoma cells and down‐regulation of TIPE2 showed reverse effects. TIPE2 overexpression increased apoptosis through down‐regulating the expression levels of Wnt3a, phospho (p)‐β‐Catenin, and p‐glycogen synthase kinase‐3β in rectal adenocarcinoma cells, however, TIPE2 knockdown exhibited reverse trends. TIPE2 overexpression decreased autophagy by reducing the expression levels of p‐Smad2, p‐Smad3, and transforming growth factor‐beta (TGF‐β) in rectal adenocarcinoma cells, however, TIPE2 knockdown showed opposite effects. Furthermore, TIPE2 overexpression reduced the growth of xenografted human rectal adenocarcinoma, whereas TIPE2 knockdown promoted the growth of rectal adenocarcinoma tumours by modulating angiogenesis. In conclusion, TIPE2 could regulate the proliferation, migration, and invasion of human rectal adenocarcinoma cells through Wnt/β‐Catenin and TGF‐β/Smad2/3 signalling pathways. TIPE2 is a potential therapeutic target for the treatment of rectal adenocarcinoma.

## 1 INTRODUCTION

Rectal cancer is one of the most common malignancies and its incidence is increasing rapidly throughout the world.^1^, ^2^, ^3^ Adenocarcinoma has become the most common histological type of rectal cancer.^4^ Rectal adenocarcinoma can be mainly stratified into four major categories, including early tumours suitable for conservative treatment, locally advanced tumours that need a major surgical procedure but are initially resectable, unresectable tumours with no distant metastases, and tumours presenting with distant metastases.^5^ Over the past several decades, surgical resection, preoperative staging technique, neoadjuvant chemoradiation therapy, and new adjuvant therapy have been widely used in the treatment of rectal adenocarcinoma.^6^, ^7^, ^8^ However, tumour recurrence and metastasis are virtually inevitable and are a major cause of death in patients with rectal adenocarcinoma.^9^ It is urgent to clarify the molecular target involved in the development of rectal adenocarcinoma, which will improve the therapeutic effect of the disease.

Tumour necrosis factor (TNF)‐α‐induced protein 8 (TNFAIP8, also known as TIPE) family consists of four members, including TNFAIP8, TIPE1, TIPE2, and TIPE3.^10^ TIPE2 is firstly identified as a gene abnormally expressed in the inflamed spinal cord of mice with experimental autoimmune encephalomyelitis.^11^ The high‐resolution crystal structure indicates that TIPE2 possesses a large, hydrophobic central cavity, which appears to be a mirror image of the death effector domain.^12^ TIPE2 is an important negative regulator of immune and inflammation homeostasis, which is closely associated with cancer development and progression.^13^, ^14^, ^15^ A number of studies have shown that TIPE2 could act as a tumour suppressor in many different types of cancer, such as osteosarcoma,^16^ gastric carcinoma,^17^ prostate carcinoma,^18^ esophageal carcinoma,^19^ hepatocellular carcinoma,^20^ and breast carcinoma.^21^ However, its roles and molecular mechanisms underlying rectal adenocarcinoma progression have not yet been elucidated.

In the present study, we initially examined the expression levels of TIPE2 in human rectal adenocarcinoma specimens. We then investigated the mechanism of action of TIPE2 on the proliferation, migration, and invasion of human rectal adenocarcinoma cells. We further examined the effects of TIPE2 on tumour growth and angiogenesis in nude mice xenografted with human rectal adenocarcinoma.

## 2 MATERIALS AND METHODS

### 2.1 Tissue samples

A total of 86 human rectal adenocarcinoma specimens and the corresponding adjacent normal tissues from rectal adenocarcinoma tissue chip (National Human Genetic Resources Sharing Service Platform, Shanghai, China) were used for the detection of TIPE2 expression by immunohistochemistry (IHC). Other four specimens were collected from patients who underwent surgery at Huaihe Hospital of Henan University, which were used for TIPE2 detection by western blot. The clinical study was approved by the Ethics Committee of Huaihe Hospital Affiliated with Henan University (2017069) and informed consent was obtained from each subject. Clinical and clinicopathological classification and staging were determined according to the American Joint Committee on Cancer criteria.^22^

### 2.2 Evaluation of immunohistochemical staining

The immunohistochemical staining results were independently evaluated by two experienced pathologists. Staining results were semiquantitatively scored based on both the staining intensity (0, negative; 1, weak; 2, moderate; 3, strong) and the percentage of positively stained cells (0, 0%; 1, 1%‐25%; 2, 26%‐50%; 3, 51%‐75%; 4, 76%‐100%). The two scores for each specimen were combined to come up with a final TIPE2 expression score. The sum of the scores were defined as follows: 0‐3, low expression; 4‐7, high expression.^23^

### 2.3 Cell culture

Human rectal adenocarcinoma cell lines HR8348 and SW837 were purchased from Shanghai HonSun Biological Technology Co., Ltd. (Shanghai, China) and Shanghai EK‐Bioscience Biotechnology Co., Ltd. (Shanghai, China), respectively. HR8348 cells were cultured in RPMI1640 medium supplemented with 10% foetal bovine serum (FBS), 100 U/mL penicillin, and 100 µg/mL streptomycin. SW837 cells were cultured in L15 medium supplemented with 10% FBS, 100 U/mL penicillin, and 100 µg/mL streptomycin. Cells were grown in an incubator with a humidified atmosphere of 95% air and 5% CO_2_ at 37˚C.

### 2.4 Overexpression and knockdown of TIPE2

Human TIPE2 complementary deoxyribonucleic acid (cDNA) (NM_024575) was subcloned into the Xho and Kpn restrictive sites of GV230 (Genechem, Shanghai, China), validated by sequencing and transfected into tumour cells with Lipofectamine 3000 Transfection Reagent (Life Technologies, Carlsbad, CA, USA). The empty vector (Mock group) or GV230‐TIPE2 construct (TIPE2 group) was transfected into tumour cells, and stable cell lines were screened by administration of G418 (Solarbio, Shanghai, China). The oligonucleotides encoding short hairpin ribonucleic acid (shRNA) specific for TIPE2 and their scramble sequences were sub‐cloned into the BamHⅠ and Hind restrictive sites of GV102 (Genechem, Shanghai, China). The TIPE2 shRNA (sh‐TIPE2 group) and scramble shRNA (sh‐Scb group) were verified by DNA sequencing and transfected into tumour cells with Lipofectamine 3000 Transfection Reagent. Stable tumour cell lines transfected with shRNAs were screened by administration of G418 (Solarbio, Shanghai, China). The untransfected tumour cells served as controls. Seventy‐two hours post‐transfection, the localization of TIPE2 within tumour cells was observed with a fluorescent microscope (Eclipse Ti, Nikon, Melville, NY, USA).

### 2.5 Reverse transcription‐polymerase chain reaction (RT‐PCR)

Total RNA was extracted from cells using TRIzol reagent, treated with DNase, and purified using an RNA clean‐up kit (Cwbiotech, Beijing, China). Total RNA (1 μg) was reverse transcribed to generate cDNAs by using a cDNA reverse transcription kit (Cwbiotech, Beijing, China). According to the primer design principles, the primers for TIPE2 were designed: forward 5’‐GTGACTGACCACATACCCCA‐3’ and reverse 5’‐AGTGTTAGTGCCAGGTGAGC‐3’. PCR reactions were performed in a total volume of 20 μL using the cycling parameters: 95°C for 10 minutes, 40 cycles of 95°C for 15 seconds, 60°C for 60 seconds, and 72°C for 1 minute. The results were normalized to the level of glyceraldehyde‐3‐phosphate dehydrogenase (GAPDH).

### 2.6 Cell proliferation and viability assays

The 5‐ethynyl‐2’‐deoxyuridine (EdU) staining assay was performed using the Cell‐Light EdU Apollo 567 In Vitro Imaging Kit (RiboBio, Guangzhou, Guangdong, China) according to the manufacturer's protocols. Then the cells were observed under a fluorescent microscope (Eclipse Ti, Nikon, Melville, NY, USA). Cell proliferation rate (%) = (EdU‐positive cells)/(total number of cells) × 100.^24^ Cell viability was determined using the CellTiter 96 AQ_ueous_ One Solution Cell Proliferation Assay kit (MTS; Promega, Madison, WI, USA) according to the manufacturer's protocols.

### 2.7 Colony formation assay

Cells were seeded in 6‐well plates at a density of 5 × 10^2^ cells per well and cultivated in culture medium for 2 weeks. The colonies were washed with phosphate‐buffered saline (PBS) buffer for three times before subjected to cell fixation using methanol (1 mL) for 15 minutes at room temperature. Then, 1 mL of crystal violet was added into each well and incubated for 30 minutes at room temperature. Plates were gently washed with deionized water thoroughly and dried in air at room temperature. Finally, plates were scanned for counting the number of colonies.

### 2.8 Wound healing assay

Confluent cells were scratched with a sterile micropipette tip to create a wound and washed twice with PBS. The migration distance was observed and photographed under an Olympus CKX41 microscope and then measured using Image J software (National Institute for Health, Bethesda, MD, USA). The migration rate (MR) was calculated as MR (%) = [(A–B)/A] × 100, where A is the width at 0 hour, and B is the width at 24 hours.^25^

### 2.9 Soft agar assay

Cells were suspended in 0.6% agarose and medium containing 10% FBS, and the mixture was seeded in 6‐well plates (1 × 10^4^ cells/well) containing a basal layer of 1.2% agarose. The medium was replaced twice a week. After two weeks, colonies were photographed under an Olympus CKX41 microscope. Colonies larger than 0.1 mm in diameter were counted.

### 2.10 Migration and invasion assays

For migration and invasion assays, 1 × 10^5^ cells were seeded in serum‐free medium and added on the upper chamber uncoated or coated with Matrigel (BD Biosciences, San Jose, CA, USA). In the lower chamber, 500 µL medium containing 10% FBS was added. After incubation for 24 hours, the remaining cells were scrubbed off with cotton swabs, while cells on the bottom surface of the membrane were fixed with 4% paraformaldehyde and then stained with 0.1% crystal violet. The cell number was counted under a Zeiss Axioskop 2 plus microscope (Carl Zeiss, Thornwood, NY, USA).

### 2.11 TdT‐mediated dUTP‐biotin nick end labelling assay

TdT‐mediated dUTP‐biotin nick end labelling (TUNEL) staining was performed using an In Situ Cell Death Detection Kit (Beyotime Biotechnology, Shanghai, China) according to the manufacturer's protocols. The cells were observed under a fluorescent microscope (Eclipse Ti, Nikon, Melville, NY, USA). The percentage of TUNEL‐positive cells was measured using Image J software.

### 2.12 Western blotting

Total protein was extracted from HR8348 and SW837 cells. Western blotting was performed to detect the expression of target proteins. The primary antibodies including anti‐β‐catenin, anti‐phospho (p)‐β‐catenin (Ser552), anti‐glycogen synthase kinase‐3 beta (Gsk‐3β), anti‐p‐Gsk‐3β (Ser9), anti‐Wnt3a, anti‐Smad2, anti‐p‐Smad2 (Ser465/467), anti‐Smad3, anti‐p‐Smad3 (Ser423/425), anti‐transforming growth factor‐beta (TGF‐β), anti‐LC3A/B, anti‐Beclin‐1, and anti‐P62 antibodies were purchased from Cell Signaling Technology (CST, Danvers, MA, USA). Anti‐TIPE2 antibody was purchased from Abcam (Cambridge, UK). Anti‐cleaved caspase‐3, anti‐cleaved poly adenosine diphosphate‐ribose polymerase (PARP), and anti‐GAPDH antibodies were purchased from ProteinTech (Chicago, IL, USA). The horseradish peroxidase‐conjugated secondary antibody was purchased from CST. The reaction was visualized using an enhanced chemiluminescence system (Thermo Fisher Scientific, Rockford, IL, USA). The bands were semi‐quantified using Image J software. The results were normalized to the level of GAPDH.

### 2.13 Animal study

Animal experiments were approved by the Committee of Medical Ethics and Welfare for Experimental Animals of Henan University School of Medicine (HUSOM‐2017‐188) in compliance with the Experimental Animal Regulations formulated by the National Science and Technology Commission, China. Animal studies were conducted as previously described with slight modifications.^26^ Thirty 4‐week‐old male BALB/C nude mice (n = 6 per group) were obtained from Beijing HFK Bioscience Co., Ltd. (Certificate No. SCXK (Jing) 2014‐0004, Beijing, China). HR8348 and SW837 cells (2 × 10^6^ cells in 200 μL PBS) with overexpression and knockdown of TIPE2 were subcutaneously inoculated into the right flanks of mice. The tumour volumes and bodyweights of mice were measured daily during the experiment. The tumour volumes were calculated as volume = L × W^2^/2, where L is the longest dimension parallel to the skin surface and W is the dimension perpendicular to L and parallel to the surface.^27^ The tumour volume doubling time (TVDT) was further calculated as TVDT = (T – T_0_) × log2/log(V2/V1), where (T – T_0_) represents the time interval and V2 and V1 indicate the volumes of tumour at the two measurement times.^28^ At the end of the experiment, the mice were sacrificed and tumours were removed and weighted to determine the inhibition rate (IR). The IR of tumour growth was calculated as IR (%) = [(A ‐ B)/A] × 100, where A is the average tumour weight of the control group, and B is that of the treatment group.^26^

### 2.14 Hematoxylin and eosin staining

After the mice were sacrifice, a necropsy examination was immediately performed. Tumour samples were fixed in 10% neutral buffered formalin, embedded in paraffin, sectioned at 5 μm thickness, and stained with hematoxylin and eosin (HE). Tumour tissues were observed with a Zeiss Axioskop 2 plus microscope.

### 2.15 IHC and evaluation

Tumour sections were stained with anti‐Ki67 antibody (CST, Danvers, MA, USA) and Ki67‐positive cells were observed under a Zeiss Axioskop 2 plus microscope. The proliferation index (PI) was calculated by the percentage of the Ki67 positive cells out of the total number of tumour cells.^29^ Cluster of differentiation 31 (CD31) has been identified as an ideal biomarker for vascular endothelial cells, and its immunostaining density is considered the tumour microvessel density (MVD).^30^ Then tumour sections were stained by IHC using CD31 antibody (CST, Danvers, MA, USA) to determine the tumour MVD. Stained vessels with a clearly defined lumen or well‐defined linear vessel shape were photographed using a Zeiss Axioskop 2 plus microscope and counted from the representative tumour zone.

### 2.16 Statistical analysis

Data are presented as mean ± SEM. The differences between multiple groups were analysed by one‐way analysis of variance using SPSS 17.0 software, followed by Tukey's test. A *P* value of less than 0.05 was considered to be statistically significant.

## 3 RESULTS

### 3.1 TIPE2 protein expression is up‐regulated in human rectal adenocarcinoma tissues compared with adjacent normal tissues

**Figure 1:**
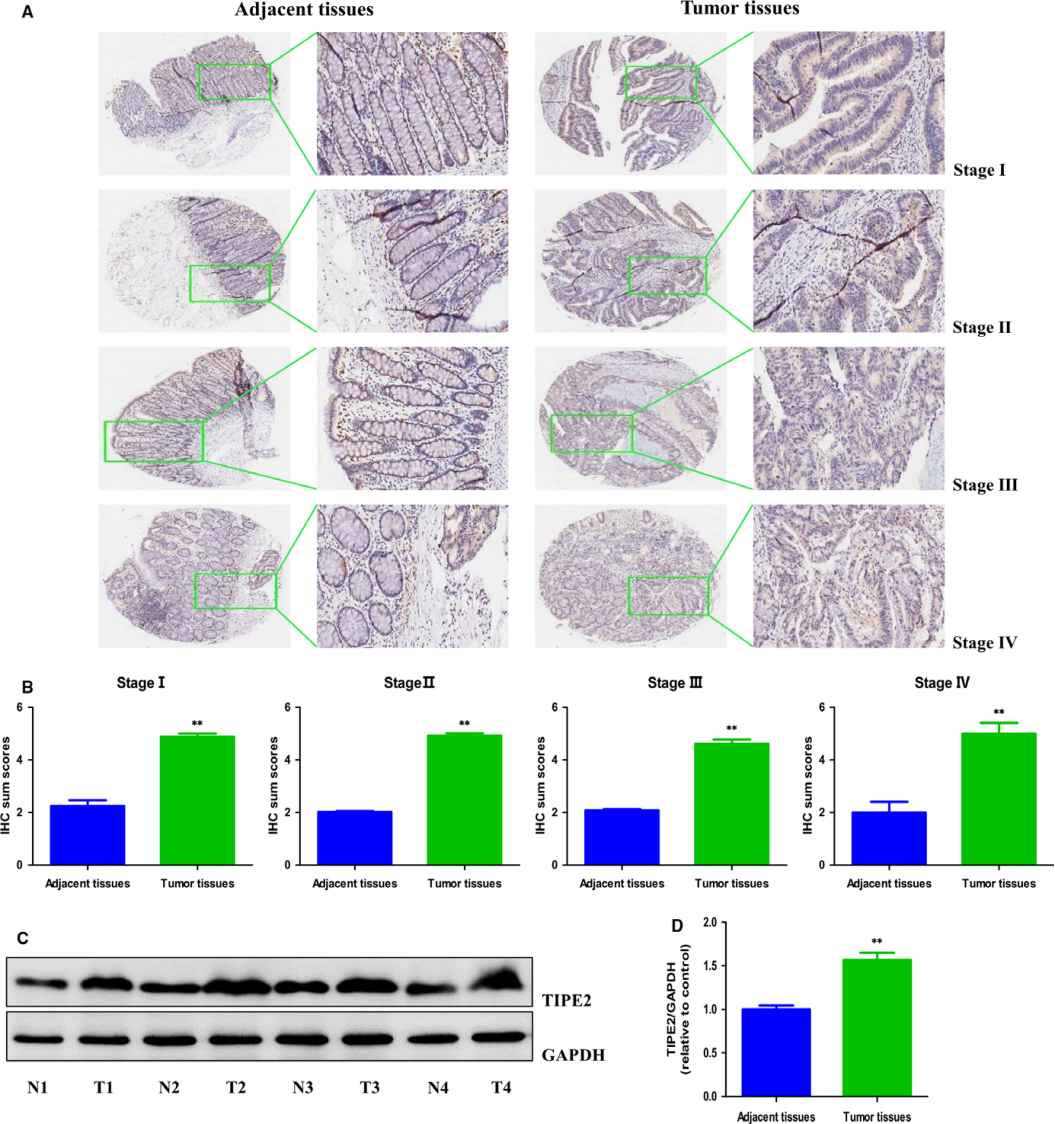
The expression of TIPE2 in human rectal adenocarcinoma tissues. A, IHC results of TIPE2 expression in different clinical stages of human rectal adenocarcinoma tissues and adjacent tissues (left: 400×; right: enlarged). B, IHC sum scores were adopted to compare TIPE2 expression in different clinical stages of human rectal adenocarcinoma tissues and adjacent tissues. C, Representative results of TIPE2 protein expression in fresh human rectal adenocarcinoma tissues (T) and adjacent normal tissues (N) detected by Western blotting. GAPDH was used as the loading control. D, Statistical results showed that the protein levels of TIPE2 were significantly elevated in fresh human rectal adenocarcinoma tissues compared to adjacent normal tissues. *P < 0.05, **P < 0.01 compared with adjacent normal tissues.

As rectal adenocarcinoma accounts for the majority of rectal cancer, we focus on rectal adenocarcinoma in this study. To explore the expression of TIPE2 protein in human rectal adenocarcinoma tissues, we detected TIPE2 expression in human rectal adenocarcinoma tissue chip that consists of 86 rectal adenocarcinoma specimens and corresponding adjacent tissues by IHC. The results showed that comparing to adjacent tissues, TIPE2 protein was highly expressed in all clinical stages of human rectal adenocarcinoma (Figure 1A and B). Then we detected the expression of TIPE2 protein in rectal adenocarcinoma fresh specimens, as well as the corresponding adjacent normal tissues (Figure 1C and D), the results further proved the aforementioned conclusions that TIPE2 expression was high in rectal adenocarcinoma tissues and low in adjacent non‐tumour tissues. To investigate the clinical significance of TIPE2 in human rectal adenocarcinoma, we further analysed the association of TIPE2 expression to clinicopathological parameters in rectal adenocarcinoma tissue chip (Table 1). Interestingly, TIPE2 expression was found to be associated with disease grade of rectal adenocarcinoma. All these data suggest that TIPE2 could serve as a promising biomarker for the diagnosis and prognosis of rectal adenocarcinoma and may act as a growth regulator in human rectal adenocarcinoma cells.

**Table 1:** Association between TIPE2 expression and clinicopathological characteristics of patients with rectal adenocarcinoma (n = 86)

Characteristics	Cases	TIPE2 expression		*P* value
		Low	High	
Age (years)				0.976
≤44	5	3	2	
45‐59	27	15	12	
≥60	54	19	35	
Gender				0.930
Male	56	21	35	
Female	30	16	14	
Tumour size (cm)				0.259
≤3	8	1	7	
>3	78	36	42	
Disease grade				0.047
I	2	1	1	
II	55	16	39	
III	29	20	9	
T classification				0.277
T1	1	1	0	
T2	10	5	5	
T3	70	30	40	
T4	5	1	4	
Lymph node status				0.873
N0	47	20	27	
N1、N2	39	17	22	
Metastasis				0.753
M0	82	35	47	
M1	4	2	2	

### 3.2 TIPE2 mediates the proliferation and viability of human rectal adenocarcinoma cells

**Figure 2:**
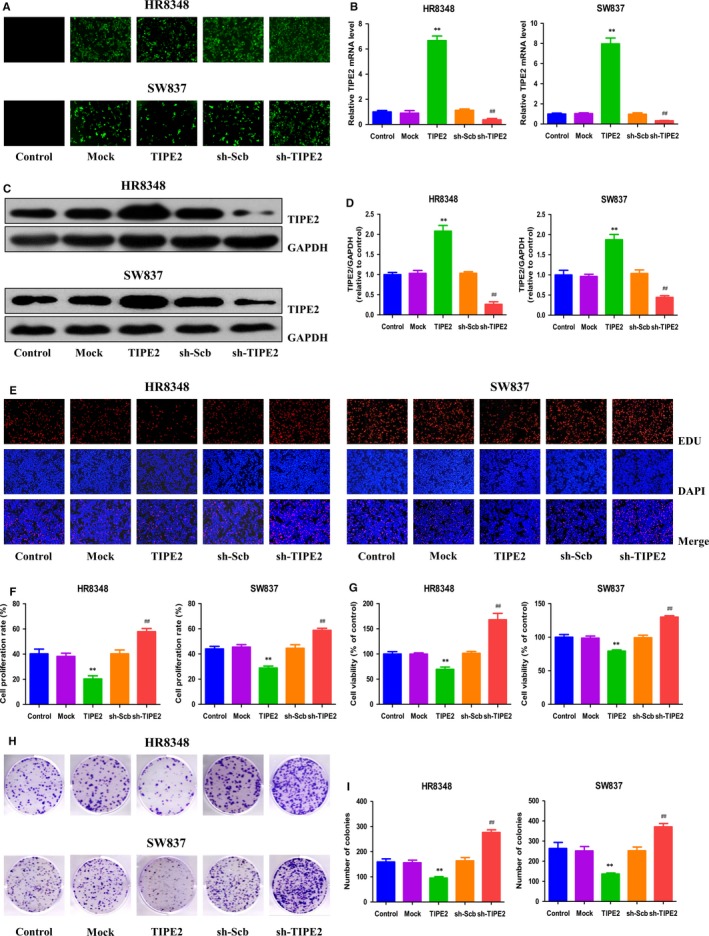
Effects of TIPE2 on the proliferation and viability of human rectal adenocarcinoma cells. A, Fluorescence microscopy of TIPE2 in HR8348 and SW837 cells; original magnification 100×. B, The expression level of TIPE2 mRNA was examined by RT‐PCR. C, The protein expression of TIPE2 was examined by Western blotting. GAPDH was used as the loading control. D, The densitometry analysis of TIPE2 was performed, normalized to the corresponding GAPDH level. E, DNA replication activities of HR8348 and SW837 cells in each group were examined by EdU assay; original magnification 100×. F, The proliferation rate of each group was analysed. G, The percentages of viable cells were determined using MTS assay and the cell viability of the control group was taken as 100%. H, The clonogenic capacity was determined in HR8348 and SW837 cells. I, The numbers of colonies were calculated. Data are presented as mean ± SEM of three independent experiments; *P < 0.05, **P < 0.01 compared with the Mock group; ^#^ *P* < 0.05, ^##^ *P* < 0.01 compared with the sh‐Scb group.

To further determine the effects of TIPE2 on the growth of human rectal adenocarcinoma cells, TIPE2 overexpression and knockdown experiments were conducted. Transfection of TIPE2 into HR8348 and SW837 cells resulted in increased expression of TIPE2 and transfection of sh‐TIPE2 decreased the expression of TIPE2 in both HR8348 and SW837 cells (Figure 2A). Furthermore, the mRNA and protein levels of TIPE2 showed similar trends (Figure 2B‐D). The results suggest that TIPE2 overexpression and knockdown experiments have been successfully conducted. As shown in Figure 2E and F, compared with the Mock group, TIPE2 overexpression reduced the proliferation of HR8348 and SW837 cells. However, TIPE2 knockdown exhibited opposite effect compared with the sh‐Scb group. TIPE2 showed similar effect on the viability of human rectal adenocarcinoma cells (Figure 2G). In addition, overexpression of TIPE2 decreased the colony formation and TIPE2 knockdown significantly increased the number of colonies (Figure 2H and I). All these results reveal that TIPE2 could mediate the proliferation and viability of human rectal adenocarcinoma cells.

### 3.3 TIPE2 mediates the migration and invasion of human rectal adenocarcinoma cells

**Figure 3:**
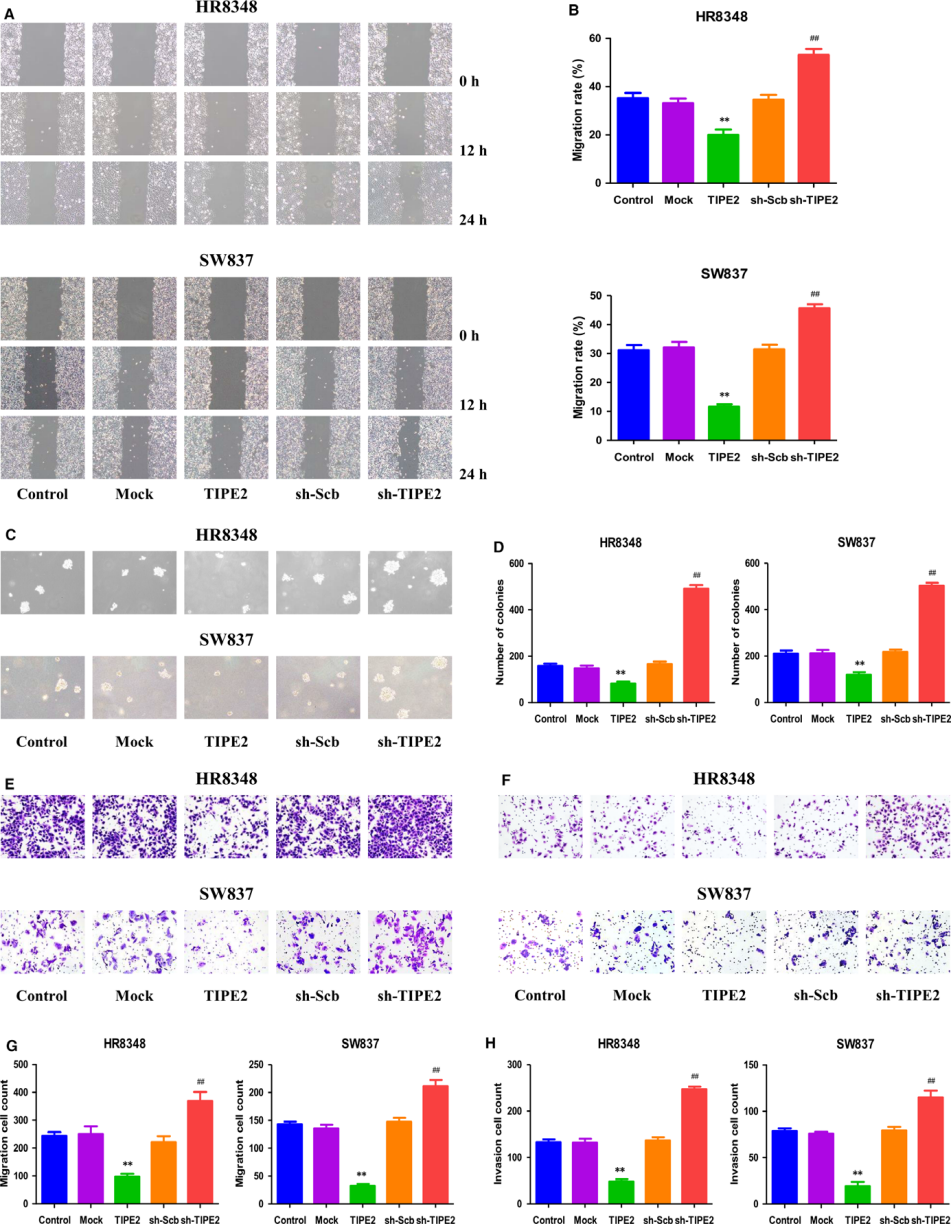
Effects of TIPE2 on the migration and invasion of human rectal adenocarcinoma cells. A, The effect of TIPE2 on cell migration was measured by wound healing assay; original magnification 100×. B, The migration rates of HR8348 and SW837 cells were calculated by the formula shown above. C, Soft agar assay was performed to examine the anchorage‐independent survival of cells; original magnification 100×. D, The number of colonies was calculated. E, Transwell assay was performed to assess the migration of HR8348 and SW837 cells; original magnification 200×. F, Transwell assay was performed to assess the invasion of HR8348 and SW837 cells; original magnification 200×. G, The numbers of the migrated cells were calculated. H, The numbers of the invasive cells were calculated. Data are presented as mean ± SEM of three independent experiments; *P < 0.05, **P < 0.01 compared with the Mock group; ^#^ *P* < 0.05, ^##^ *P* < 0.01 compared with the sh‐Scb group.

In scratch migration assay, TIPE2 overexpression reduced the migration capabilities of HR8348 and SW837 cells and TIPE2 knockdown showed reverse trends (Figure 3A and B). In soft agar assay, overexpression of TIPE2 attenuated the anchorage‐independent growth of HR8348 and SW837 cells, whereas reverse effects were observed in sh‐TIPE2 group (Figure 3C and D). Transwell analysis showed that the migration and invasion capacities of HR8348 and SW837 cells were reduced in TIPE2 group, while the sh‐TIPE2 group exhibited reverse trends (Figure 3E‐H). Taken together, these results show that TIPE2 plays an important role in regulating the migration and invasion of human rectal adenocarcinoma cells.

### 3.4 TIPE2 modulates apoptosis through the Wnt/β‐Catenin signalling pathway in human rectal adenocarcinoma cells

**Figure 4:**
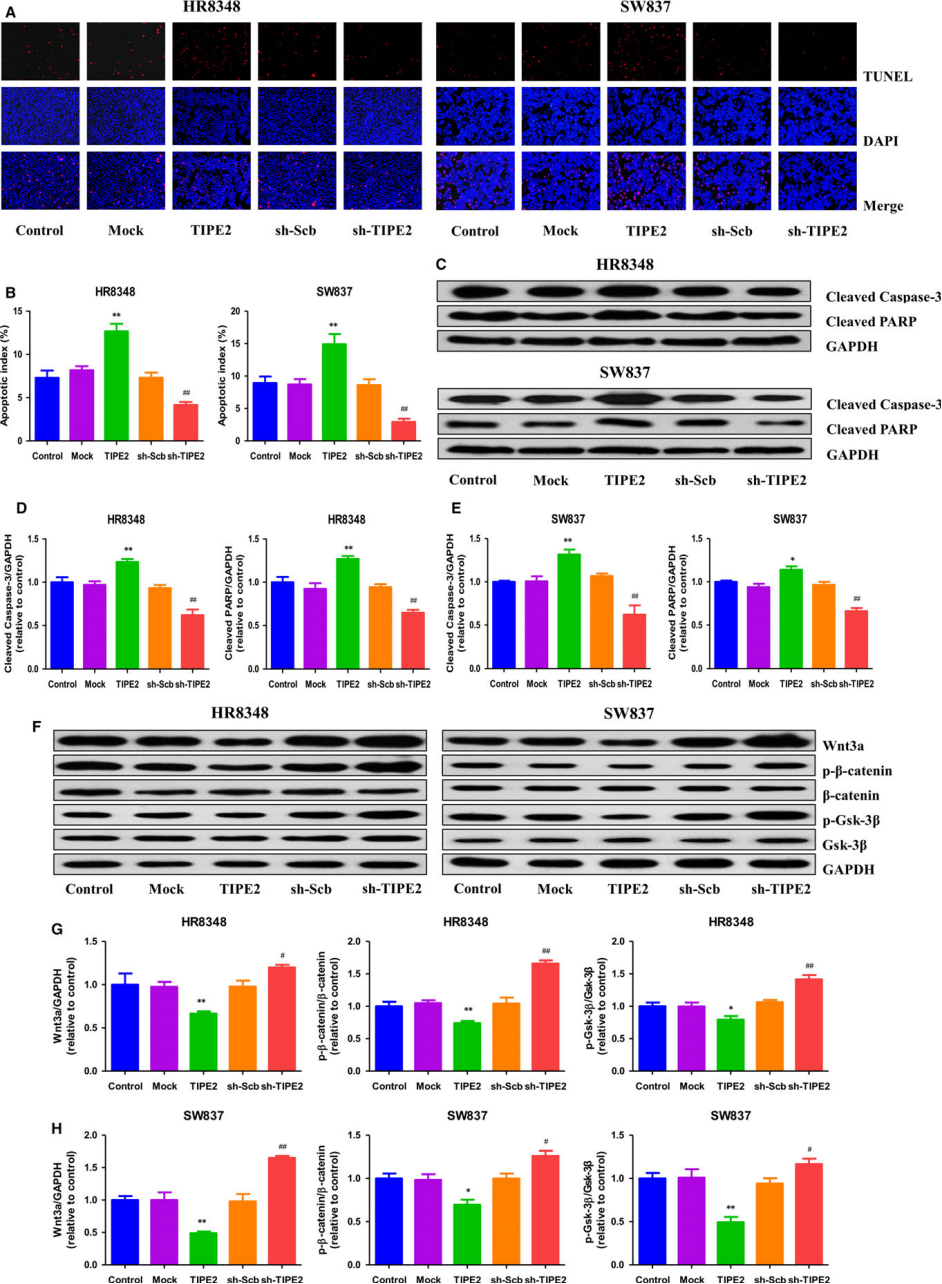
Effects of TIPE2 on the apoptosis and Wnt/β‐Catenin signalling pathway in human rectal adenocarcinoma cells. A, The apoptotic levels of HR8348 and SW837 cells were measured by TUNEL staining; original magnification 100×. B, The percentages of TUNEL‐positive cells were calculated by the formula shown above. C, Western blotting analysis for the expression of cleaved caspase‐3 and cleaved PARP in HR8348 and SW837 cells. GAPDH was used as the loading control. D, E, The densitometry analyses of cleaved caspase‐3 and cleaved PARP were performed in HR8348 and SW837 cells, normalized to the corresponding GAPDH level. F, Western blotting analysis for the expression of Wnt3a, p‐β‐catenin, β‐catenin, p‐Gsk‐3β, and Gsk‐3β in HR8348 and SW837 cells. GAPDH was used as the loading control. G, H The densitometry analyses of Wnt3a, p‐β‐catenin, β‐catenin, p‐Gsk‐3β, and Gsk‐3β were performed in HR8348 and SW837 cells, normalized to the corresponding GAPDH level. Data are presented as mean ± SEM of three independent experiments; *P < 0.05, **P < 0.01 compared with the Mock group; ^#^ *P* < 0.05, ^##^ *P* < 0.01 compared with the sh‐Scb group.

As shown in Figure 4A and B, the apoptotic index increased in the TIPE2 group compared with the Mock group and decreased in the sh‐TIPE2 group compared with the sh‐Scb group. In addition, the protein levels of cleaved caspase‐3 and cleaved PARP in human rectal adenocarcinoma cells exhibited similar trends (Figure 4C‐E). It has been shown that the Wnt/β‐Catenin pathway regulates the expression of a number of genes involved in apoptosis and GSK‐3β plays an important role in Wnt/β‐Catenin signalling pathway.^31^, ^32^. As shown in Figure 4F‐H, the expression levels of Wnt3a, p‐β‐Catenin, and p‐Gsk‐3β in the TIPE2 group were lower than those in the Mock group, however, the expression levels of these proteins in the sh‐TIPE2 group were higher than those in the sh‐Scb group. These results together suggest that TIPE2 could modulate apoptosis through the Wnt/β‐Catenin signalling pathway in human rectal adenocarcinoma cells.

### 3.5 TIPE2 modulates autophagy through the TGF‐β/Smad2/3 signalling pathway in human rectal adenocarcinoma cells

**Figure 5:**
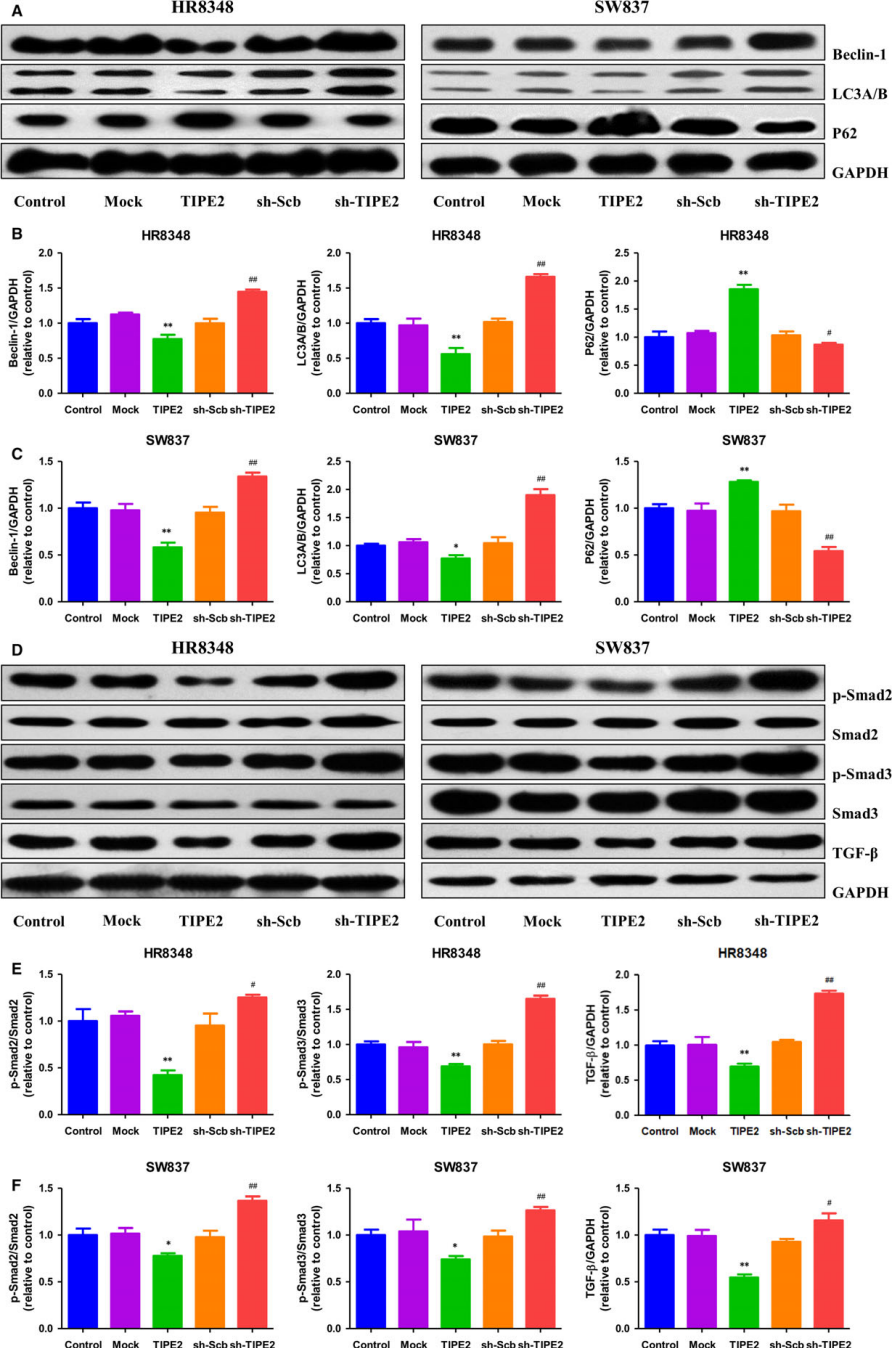
Effects of TIPE2 on the autophagy and TGF‐β/Smad2/3 signalling pathway in human rectal adenocarcinoma cells. A, Western blotting analysis for the expression of Beclin‐1, LC3A/B, and P62 in HR8348 and SW837 cells. GAPDH was used as the loading control. B, C, The densitometry analyses of Beclin‐1, LC3A/B, and P62 were performed in HR8348 and SW837 cells, normalized to the corresponding GAPDH level. D, Western blotting analysis for the expression of p‐Smad2, Smad2, p‐Smad3, Smad3, and TGF‐β in HR8348 and SW837 cells. GAPDH was used as the loading control. E, F, The densitometry analyses of p‐Smad2, Smad2, p‐Smad3, Smad3, and TGF‐β were performed in HR8348 and SW837 cells, normalized to the corresponding GAPDH level. Data are presented as mean ± SEM of three independent experiments; *P < 0.05, **P < 0.01 compared with the Mock group; ^#^ *P* < 0.05, ^##^ *P* < 0.01 compared with the sh‐Scb group.

Autophagy is a multistep cellular process by which cellular materials are delivered to lysosomes for degradation and recycling.^33^ Autophagy is involved in cellular homeostasis, development, physiology, and abnormalities in autophagy may result in many pathophysiological conditions.^34^ LC3, Beclin 1, and p62 play key roles in the process of autophagy and have been considered specific autophagic markers.^35^, ^36^ As shown in Figure 5A‐C, the protein levels of Beclin 1 and LC3 in the TIPE2 group were lower than those in the Mock group, whereas the expression levels of these two proteins were higher in the sh‐TIPE2 group than those in the sh‐Scb group. Furthermore, the expression levels of P62 showed reverse trends. TGF‐β is an important regulator of the autophagy in a variety of cell types.^37^ As canonical effectors of TGF‐β signalling, Smad2/3 have also been shown to control autophagy.^38^ As shown in Figure 5D‐F, the protein levels of p‐Smad2, p‐Smad3, and TGF‐β in the TIPE2 group were lower than those in the Mock group, whereas the expression levels of these proteins in the sh‐TIPE2 group were higher than those in the sh‐Scb group. Collectively, the results indicate that TIPE2 could modulate autophagy through the TGF‐β/Smad2/3 signalling pathway in human rectal adenocarcinoma cells.

### 3.6 TIPE2 regulates the growth and angiogenesis of human rectal adenocarcinoma xenograft tumours in nude mice

**Figure 6:**
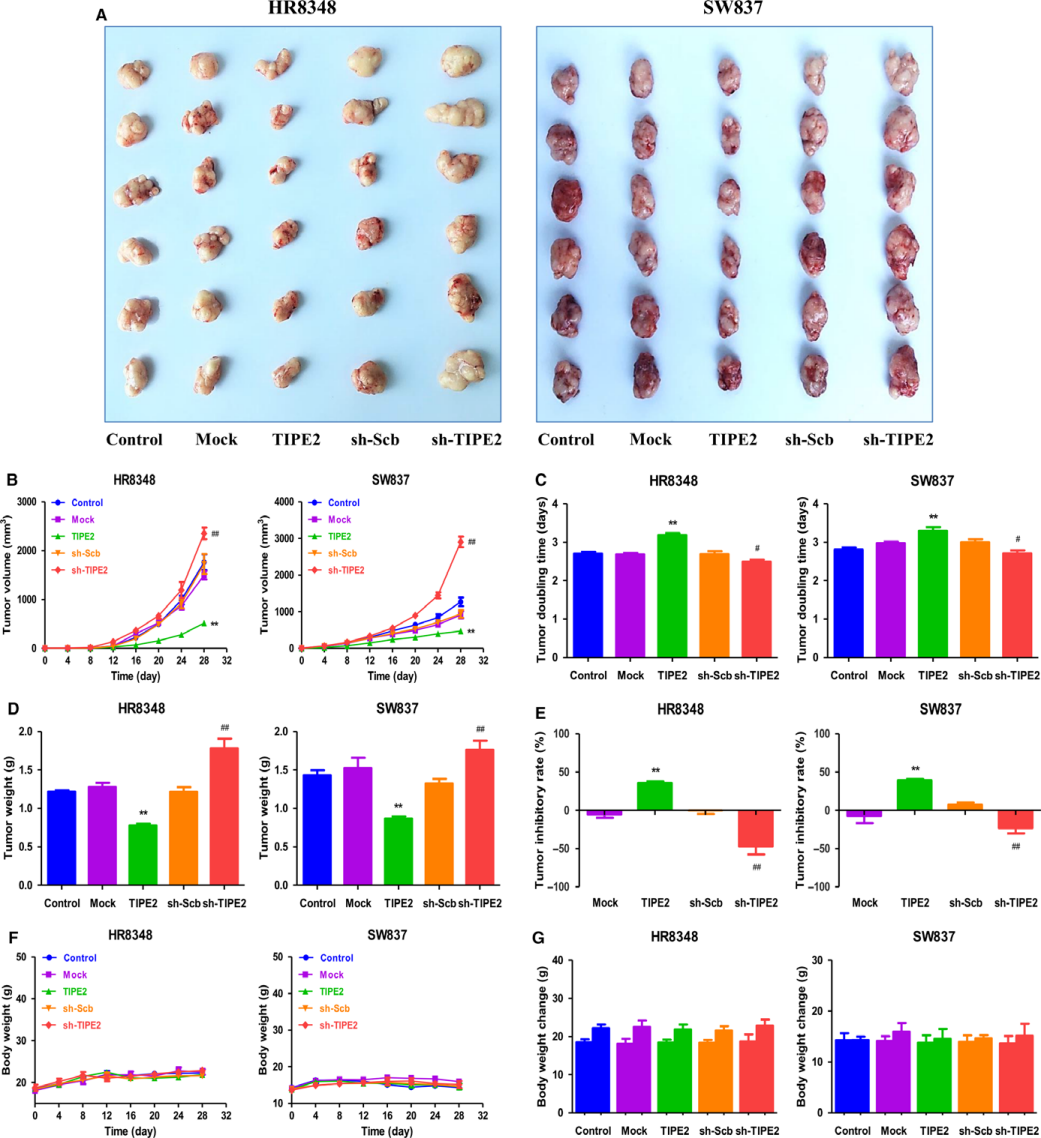
Effects of TIPE2 on the growth of HR8348 and SW837 xenograft tumours in nude mice. A, Representative xenografts dissected from different groups of nude mice were shown. B, C, The tumour volume of each group was measured every day and the TVDT was calculated by the formula shown above. D, E, The tumours were weighed and the inhibition rates of tumour growth were calculated by the formula shown above. F, G, The bodyweight change curve of each group during the experiment and the bodyweight of each group on the first day (day 0) and the last day (day 28). Values are presented as mean ± SEM (n = 6); *P < 0.05, **P < 0.01 compared with the Mock group; ^#^ *P* < 0.05, ^##^ *P* < 0.01 compared with the sh‐Scb group.

**Figure 7:**
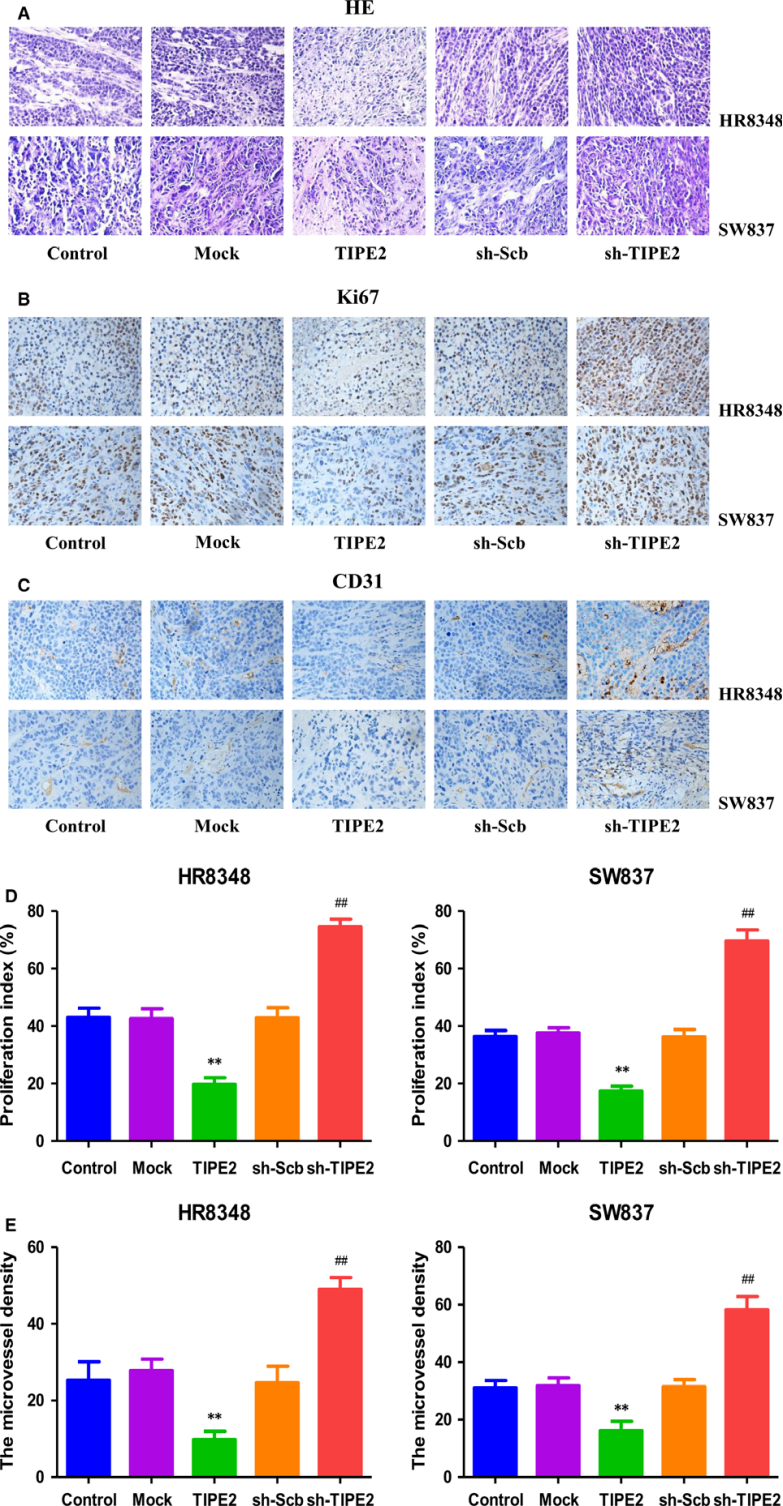
Effects of TIPE2 on the PI and MVD of human rectal adenocarcinoma xenografts. A‐C, Representive photographs of HE, Ki67, and CD31 staining in HR8348 and SW837 xenograft tumours; original magnification 400×. D, E, The PI and MVD were calculated by the formula shown above. Values are presented as mean ± SEM (n = 6); *P < 0.05, **P < 0.01 compared with the Mock group; ^#^ *P* < 0.05, ^##^ *P* < 0.01 compared with the sh‐Scb group.

HR8348 and SW837 cells have been adopted to establish subcutaneous xenograft models.^39^, ^40^ Then the effects of TIPE2 on the growth of rectal adenocarcinoma xenograft tumours were determined. TIPE2 overexpression reduced the growth of xenograft tumours, when compared to the Mock group. In contrast, TIPE2 knockdown promoted the growth of xenograft tumours compared with the sh‐Scb group (Figures 6A‐E). Moreover, there was no significant difference in bodyweight between each group (Figure 6F and G). IHC with the Ki67 antibody demonstrated that the in vivo proliferation of rectal adenocarcinoma cells was decreased in the TIPE2 group compared with the Mock group and increased in the sh‐TIPE2 group compared with the sh‐Scb group. In addition, the protein expression of CD31 in rectal adenocarcinoma xenograft tumours showed a similar trend (Figure 7). In sum, these results show that TIPE2 could mediate the growth and angiogenesis of human rectal adenocarcinoma xenograft tumours.

## 4 DISCUSSION

TIPE2 has recently been considered a key negative regulator of immune and inflammation homeostasis, which is closely associated with cancer development and progression.^13^, ^14^, ^15^ The expression levels of TIPE2 in normal tissues and tumours are of high specificity in both humans and mice. It has been demonstrated that TIPE2 protein expression is very weak in rectum and is not detected or weakly expressed in most human carcinoma cell lines.^41^, ^42^ Another study has shown that TIPE2 expression is higher in colon cancer tissues compared to normal controls and it is related with lymph node metastasis and Dukes stage of colon cancer.^43^ Our results indicated that TIPE2 expression was higher in rectal adenocarcinoma tissues compared to adjacent nontumour tissues. In addition, TIPE2 expression was found to be associated with disease grade of rectal adenocarcinoma. Based on these findings, we can draw the conclusion that TIPE2 expression is high in colorectal cancer tissues and low in adjacent nontumour tissues, suggesting that TIPE2 could be a promising biomarker for the diagnosis and prognosis of colorectal cancer and may play a role in the development and procession of colorectal cancer.

An increasing number of studies have demonstrated that TIPE2 is a tumour suppressor in many types of cancer.^16^, ^17^, ^18^, ^19^, ^20^, ^21^ However, the mechanisms of action of TIPE2 on the growth of rectal adenocarcinoma are still unknown. The human rectal adenocarcinoma cell lines HR8348 and SW837 have been widely used as cellular models to study the intracellular mechanisms of action of therapeutic agents.^39^, ^40^, ^44^ In the present study, HR8348 and SW837 cells were used to evaluate the effects of TIPE2 both in vitro and in vivo and TIPE2 overexpression and knockdown experiments were performed. A recent study has shown that TIPE2 overexpression could induce cell death and inhibit Ras‐mediated tumourigenesis in mice.^10^ In addition, deregulation of TIPE2 is found to promote the essential hallmarks of cancer such as survival, proliferation, invasion, migration, and metastasis.^15^ Similarly, our results showed that TIPE2 overexpression reduced the proliferation and viability, as well as decreased the migration and invasion capabilities of HR8348 and SW837 cells, whereas TIPE2 knockdown exhibited completely opposite effects, indicating that TIPE2 plays important roles in the growth, migration, and invasion of human rectal adenocarcinoma cells.

Apoptosis, or programmed cell death, is a critical process for the development and maintenance of cellular homeostasis in multicellular organisms.^45^ There are two major pathways leading to apoptosis in the mammalian system: an intrinsic pathway that occurs through the mitochondria and an extrinsic pathway initiated by death receptors.^46^ Caspases can be activated in response to diverse apoptotic stimuli and PARP is further cleaved by activated caspase‐3, thus leading to the occurrence of apoptotic cascade.^26^ It has been reported that the adenovirus expressing human TIPE2 could obviously up‐regulate the expression levels of Bcl‐2‐associated X protein, cleaved caspase‐3, 9, and PARP in AGS human gastric cancer cells.^47^ Our results showed that TIPE2 overexpression significantly increased the apoptotic index and protein expressions of cleaved caspase‐3 and PARP, whereas TIPE2 knockdown remarkably decreased the apoptotic levels, suggesting that TIPE2 could mediate mitochondria‐mediated pathway in human rectal adenocarcinoma cells. Wnt/β‐Catenin pathway regulates the expression of many genes involved in apoptosis and GSK‐3β plays a key role in Wnt/β‐Catenin signalling pathway.^31^, ^32^ Another study has shown that TIPE2 could suppress progression and tumourigenesis of esophageal carcinoma via inhibition of the Wnt/β‐catenin pathway.^19^ Similarly, our results indicated that the expression levels of Wnt3a, p‐β‐Catenin, and p‐Gsk‐3β in the TIPE2 group were lower than those in the Mock group. However, higher expression levels of these proteins have been observed in the sh‐TIPE2 group when compared to the sh‐Scb group. Therefore, we can conclude that TIPE2 modulates apoptosis through the Wnt/β‐Catenin signalling pathway in human rectal adenocarcinoma cells.

Autophagy is a highly conserved evolutionary process by which cellular materials are delivered to lysosomes for degradation, resulting in the basal turnover of cell components and providing energy and macromolecular precursors.^33^, ^48^ In some contexts, autophagy can suppress tumourigenesis, whereas in most contexts autophagy facilitates tumourigenesis.^49^ Furthermore, it has been shown that tumours are more autophagy‐dependent than normal tissues. Therefore, autophagy inhibition may be an effective way for cancer therapy.^50^ The results indicated that TIPE2 overexpression decreased the autophagy level compared with the Mock group, and TIPE2 suppression showed reverse effects compare to the sh‐Scb group. TGF‐β, a multifunctional cytokine, could activate autophagy in many human cancer cells.^51^, ^52^, ^53^ As transcriptional mediators of TGF‐β signalling, Smad2/3 have also been shown to control autophagy.^38^, ^54^ Our results showed that TIPE2 overexpression reduced the protein levels of p‐Smad2, p‐Smad3, and TGF‐β compared to the Mock group, whereas TIPE2 knockdown promoted the expression levels of these proteins compare to the sh‐Scb group. These results demonstrate that TIPE2 could modulate autophagy through the TGF‐β/Smad2/3 signalling pathway in human rectal adenocarcinoma cells.

Recent studies indicate that HR8348 and SW837 cells have been used to establish subcutaneous xenograft models.^39^, ^40^ We therefore determined the effects of TIPE2 on the growth of rectal adenocarcinoma xenograft tumours in BALB/c nude mice. TIPE2 overexpression notably reduced the growth of rectal adenocarcinoma xenograft tumours, whereas TIPE2 knockdown markedly promoted tumour growth. Ki67, a nuclear nonhistone protein, is expressed by proliferating cells in all phases of the active cell cycle.^55^ The expression level of Ki67 is closely associated with the proliferation, invasiveness, and clinical outcome of a number of malignant tumours.^56^ Ki67 is regarded as an important proliferative marker and has been widely adopted in determining the proliferation of cancer cells.^26^, ^55^, ^56^ In line with the in vitro findings, the results showed that the expression of Ki67 was decreased in the TIPE2 group and increased in the sh‐TIPE2 group. CD31 has been considered an ideal biomarker for vascular endothelial cells and its density is generally represented by the tumour MVD.^26^, ^30^ The results indicated that TIPE2 overexpression reduced the expression of CD31, while TIPE2 knockdown promoted the expression of CD31 in rectal adenocarcinoma xenograft tumours, suggesting that TIPE2 could regulate the growth of human rectal adenocarcinoma xenograft tumours by mediating angiogenesis.

In conclusion, our results demonstrate that TIPE2 can be detected in human rectal adenocarcinoma cells and the expression levels of TIPE2 in human rectal adenocarcinoma tissues are higher than those in adjacent nontumour tissues. Furthermore, the present study indicates that TIPE2 could mediate the proliferation, migration, and invasion of human rectal adenocarcinoma cells through Wnt/β‐Catenin and TGF‐β/Smad2/3 signalling pathways. Considering its role in the developmental process of human rectal adenocarcinoma cells, TIPE2 could be a potential therapeutic target for advanced and recurrent human rectal adenocarcinoma.

## CONFLICT OF INTEREST

The authors confirm that there are no conflicts of interest.

## References

[1] Torre LA , Bray F , Siegel RL , et al. Global cancer statistics, 2012. CA Cancer J Clin. 2015;65:87‐108. 25651787 10.3322/caac.21262

[2] Siegel RL , Miller KD , Jemal A . Cancer statistics, 2015. CA Cancer J Clin. 2015;65:5‐29. 25559415 10.3322/caac.21254

[3] Sung JJ , Ng SC , Chan FK , et al. An updated Asia Pacific Consensus Recommendations on colorectal cancer screening. Gut. 2015;64:121‐132. 24647008 10.1136/gutjnl-2013-306503

[4] Hugen N , Brown G , Glynne‐Jones R , et al. Advances in the care of patients with mucinous colorectal cancer. Nat Rev Clin Oncol. 2016;13:361‐369. 26323388 10.1038/nrclinonc.2015.140

[5] Mendenhall WM , Zlotecki RA , Snead FE , et al. Radiotherapy in the treatment of resectable rectal adenocarcinoma. Am J Clin Oncol. 2009;32:629‐638. 19593081 10.1097/COC.0b013e31817ff8e4

[6] Ellis CT , Samuel CA , Stitzenberg KB . National trends in nonoperative management of rectal adenocarcinoma. J Clin Oncol. 2016;34:1644‐1651. 27022115 10.1200/JCO.2015.64.2066

[7] Garcia‐Aguilar J , Chow OS , Smith DD , et al. Effect of adding mFOLFOX6 after neoadjuvant chemoradiation in locally advanced rectal cancer: a multicentre, phase 2 trial. Lancet Oncol. 2015;16:957‐966. 26187751 10.1016/S1470-2045(15)00004-2 PMC4670237

[8] Rickles AS , Dietz DW , Chang GJ , et al. High rate of positive circumferential resection margins following rectal cancer surgery: a call to action. Ann Surg. 2015;262:891‐898. 26473651 10.1097/SLA.0000000000001391 PMC5260485

[9] Fan CW , Chen T , Shang YN , et al. Cancer‐initiating cells derived from human rectal adenocarcinoma tissues carry mesenchymal phenotypes and resist drug therapies. Cell Death Dis. 2013;4:e828. 24091671 10.1038/cddis.2013.337 PMC3824647

[10] Gus‐Brautbar Y , Johnson D , Zhang L , et al. The anti‐inflammatory TIPE2 is an inhibitor of the oncogenic Ras. Mol Cell. 2012;45:610‐618. 22326055 10.1016/j.molcel.2012.01.006 PMC3299909

[11] Sun H , Gong S , Carmody RJ , et al. TIPE2, a negative regulator of innate and adaptive immunity that maintains immune homeostasis. Cell. 2008;133:415‐426. 18455983 10.1016/j.cell.2008.03.026 PMC2398615

[12] Zhang X , Wang J , Fan C , et al. Crystal structure of TIPE2 provides insights into immune homeostasis. Nat Struct Mol Biol. 2009;16:89‐90. 19079267 10.1038/nsmb.1522

[13] Freundt EC , Bidere N , Lenardo MJ . A different TIPE of immune homeostasis. Cell. 2008;133:401‐402. 18455981 10.1016/j.cell.2008.04.017 PMC2750003

[14] Elinav E , Nowarski R , Thaiss CA , et al. Inflammation‐induced cancer: crosstalk between tumours, immune cells and microorganisms. Nat Rev Cancer. 2013;13:759‐771. 24154716 10.1038/nrc3611

[15] Padmavathi G , Banik K , Monisha J , et al. Novel tumor necrosis factor‐α induced protein eight (TNFAIP8/TIPE) family: Functions and downstream targets involved in cancer progression. Cancer Lett. 2018;432:260‐271. 29920292 10.1016/j.canlet.2018.06.017

[16] Deng B , Feng Y , Deng B . TIPE2 Mediates the suppressive Effects of Shikonin on MMP13 in osteosarcoma cells. Cell Physiol Biochem. 2015;37:2434‐2443. 26650545 10.1159/000438596

[17] Yin H , Huang X , Tao M , et al. Adenovirus‐mediated TIPE2 overexpression inhibits gastric cancer metastasis via reversal of epithelial‐mesenchymal transition. Cancer Gene Ther. 2017;24:180‐188. 28186089 10.1038/cgt.2017.3

[18] Lu Q , Liu Z , Li Z , et al. TIPE2 overexpression suppresses the proliferation, migration, and invasion in prostate cancer cells by inhibiting PI3K/Akt signaling pathway. Oncol Res. 2016;24:305‐313. 27712587 10.3727/096504016X14666990347437 PMC7838667

[19] Zhu L , Zhang X , Fu X , et al. TIPE2 suppresses progression and tumorigenesis of esophageal carcinoma via inhibition of the Wnt/β‐catenin pathway. J Transl Med. 2018;16:7. 29343267 10.1186/s12967-018-1383-0 PMC5773041

[20] Cao X , Zhang L , Shi Y , et al. Human tumor necrosis factor (TNF)‐alpha‐induced protein 8‐like 2 suppresses hepatocellular carcinoma metastasis through inhibiting Rac1. Mol Cancer. 2013;12:149. 24274578 10.1186/1476-4598-12-149 PMC4176125

[21] Zhang Z , Liu L , Cao S , et al. Gene delivery of TIPE2 inhibits breast cancer development and metastasis via CD8+ T and NK cell‐mediated antitumor responses. Mol Immunol. 2017;85:230‐237. 28314212 10.1016/j.molimm.2017.03.007

[22] Dinaux AM , Leijssen L , Bordeianou LG , et al. Outcomes of persistent lymph node involvement after neoadjuvant therapy for stage III rectal cancer. Surgery. 2018;163:784‐788. 29277386 10.1016/j.surg.2017.10.021

[23] Li Z , Guo C , Liu X , et al. TIPE2 suppresses angiogenesis and non‐small cell lung cancer (NSCLC) invasiveness via inhibiting Rac1 activation and VEGF expression. Oncotarget. 2016;7:62224‐62239. 27556698 10.18632/oncotarget.11406 PMC5308722

[24] Zhang GY , Lu D , Duan SF , et al. Hydrogen Sulfide Alleviates Lipopolysaccharide‐induced diaphragm dysfunction in rats by reducing apoptosis and inflammation through ROS/MAPK and TLR4/NF‐κB signaling pathways. Oxid Med Cell Longev. 2018;2018:9647809. 29977458 10.1155/2018/9647809 PMC5994286

[25] Wu D , Li M , Tian W , et al. Hydrogen sulfide acts as a double‐edged sword in human hepatocellular carcinoma cells through EGFR/ERK/MMP‐2 and PTEN/AKT signaling pathways. Sci Rep. 2017;7:5134. 28698660 10.1038/s41598-017-05457-z PMC5506015

[26] Wu DD , Gao YR , Li T , et al. PEST‐containing nuclear protein mediates the proliferation, migration, and invasion of human neuroblastoma cells through MAPK and PI3K/AKT/mTOR signaling pathways. BMC Cancer. 2018;18:499. 29716528 10.1186/s12885-018-4391-9 PMC5930684

[27] Heilmann AM , Perera RM , Ecker V , et al. CDK4/6 and IGF1 receptor inhibitors synergize to suppress the growth of p16INK4A‐deficient pancreatic cancers. Cancer Res. 2014;74:3947‐3958. 24986516 10.1158/0008-5472.CAN-13-2923 PMC4122288

[28] Ellingson BM , Nguyen HN , Lai A , et al. Contrast‐enhancing tumor growth dynamics of preoperative, treatment‐naive human glioblastoma. Cancer. 2016;122:1718‐1727. 26998740 10.1002/cncr.29957

[29] Keam B , Im SA , Lee KH , et al. Ki‐67 can be used for further classification of triple negative breast cancer into two subtypes with different response and prognosis. Breast Cancer Res. 2011;13:R22. 21366896 10.1186/bcr2834 PMC3219180

[30] Tolaney SM , Boucher Y , Duda DG , et al. Role of vascular density and normalization in response to neoadjuvant bevacizumab and chemotherapy in breast cancer patients. Proc Natl Acad Sci USA. 2015;112:14325‐14330. 26578779 10.1073/pnas.1518808112 PMC4655544

[31] Kahn M . Can we safely target the WNT pathway? Nat Rev Drug Discov. 2014;13:513‐532. 24981364 10.1038/nrd4233 PMC4426976

[32] Wu D , Pan W . GSK3: a multifaceted kinase in Wnt signaling. Trends Biochem Sci. 2010;35:161‐168. 19884009 10.1016/j.tibs.2009.10.002 PMC2834833

[33] Levy J , Towers CG , Thorburn A . Targeting autophagy in cancer. Nat Rev Cancer. 2017;17:528‐542. 28751651 10.1038/nrc.2017.53 PMC5975367

[34] Wu D , Wang H , Teng T , et al. Hydrogen sulfide and autophagy: A double edged sword. Pharmacol Res. 2018;131:120‐127. 29514056 10.1016/j.phrs.2018.03.002

[35] Dokladny K , Myers OB , Moseley PL . Heat shock response and autophagy–cooperation and control. Autophagy. 2015;11:200‐213. 25714619 10.1080/15548627.2015.1009776 PMC4502786

[36] Liu J , Liu W , Lu Y , et al. Piperlongumine restores the balance of autophagy and apoptosis by increasing BCL2 phosphorylation in rotenone‐induced Parkinson disease models. Autophagy. 2018;14:845‐861. 29433359 10.1080/15548627.2017.1390636 PMC6070010

[37] Ding Y , Choi ME . Regulation of autophagy by TGF‐β: emerging role in kidney fibrosis. Semin Nephrol. 2014;34:62‐71. 24485031 10.1016/j.semnephrol.2013.11.009 PMC3912517

[38] Pan CC , Kumar S , Shah N , et al. Endoglin regulation of Smad2 function mediates Beclin1 expression and endothelial autophagy. J Biol Chem. 2015;290:14884‐14892. 25931117 10.1074/jbc.M114.630178 PMC4463436

[39] Li S , Yu B , An P , et al. Combined liposome‐mediated cytosine deaminase gene therapy with radiation in killing rectal cancer cells and xenografts in athymic mice. Clin Cancer Res. 2005;11:3574‐3578. 15867262 10.1158/1078-0432.CCR-04-2077

[40] Spitzner M , Roesler B , Bielfeld C , et al. STAT3 inhibition sensitizes colorectal cancer to chemoradiotherapy in vitro and in vivo. Int J Cancer. 2014;134:997‐1007. 23934972 10.1002/ijc.28429 PMC7706351

[41] Zhang L , Shi Y , Wang Y , et al. The unique expression profile of human TIPE2 suggests new functions beyond its role in immune regulation. Mol Immunol. 2011;48:1209‐1215. 21459448 10.1016/j.molimm.2011.03.001

[42] Zhang G , Hao C , Lou Y , et al. Tissue‐specific expression of TIPE2 provides insights into its function. Mol Immunol. 2010;47:2435‐2442. 20663561 10.1016/j.molimm.2010.06.016

[43] Li XM , Su JR , Yan SP , et al. A novel inflammatory regulator TIPE2 inhibits TLR4‐mediated development of colon cancer via caspase‐8. Cancer Biomark. 2014;14:233‐240. 24934366 10.3233/CBM-140402 PMC12928344

[44] Zhang Y , Yu J , Liu H , et al. Novel epigenetic CREB‐miR‐630 signaling axis regulates radiosensitivity in colorectal cancer. PLoS ONE. 2015;10:e0133870. 26263387 10.1371/journal.pone.0133870 PMC4532457

[45] Wu D , Si W , Wang M , et al. Hydrogen sulfide in cancer: friend or foe? Nitric Oxide. 2015;50:38‐45. 26297862 10.1016/j.niox.2015.08.004

[46] Wu D , Gao Y , Qi Y , et al. Peptide‐based cancer therapy: opportunity and challenge. Cancer Lett. 2014;351:13‐22. 24836189 10.1016/j.canlet.2014.05.002

[47] Zhu Y , Tao M , Wu J , et al. Adenovirus‐directed expression of TIPE2 suppresses gastric cancer growth via induction of apoptosis and inhibition of AKT and ERK1/2 signaling. Cancer Gene Ther. 2016;23:98‐106. 26987289 10.1038/cgt.2016.6

[48] Farrow JM , Yang JC , Evans CP . Autophagy as a modulator and target in prostate cancer. Nat Rev Urol. 2014;11:508‐516. 25134829 10.1038/nrurol.2014.196 PMC4415606

[49] White E . The role for autophagy in cancer. J Clin Invest. 2015;125:42‐46. 25654549 10.1172/JCI73941 PMC4382247

[50] White E , Mehnert JM , Chan CS . Autophagy, metabolism, and cancer. Clin Cancer Res. 2015;21:5037‐5046. 26567363 10.1158/1078-0432.CCR-15-0490 PMC4646728

[51] Jiang Y , Woosley AN , Sivalingam N , et al. Cathepsin‐B‐mediated cleavage of Disabled‐2 regulates TGF‐β‐induced autophagy. Nat Cell Biol. 2016;18:851‐863. 27398911 10.1038/ncb3388 PMC5937135

[52] Zhang C , Zhang X , Xu R , et al. TGF‐β2 initiates autophagy via Smad and non‐Smad pathway to promote glioma cells' invasion. J Exp Clin Cancer Res. 2017;36:162. 29145888 10.1186/s13046-017-0628-8 PMC5689187

[53] Nüchel J , Ghatak S , Zuk AV , et al. TGFB1 is secreted through an unconventional pathway dependent on the autophagic machinery and cytoskeletal regulators. Autophagy. 2018;14:465‐486. 29297744 10.1080/15548627.2017.1422850 PMC5915026

[54] Kumar S , Pan CC , Shah N , et al. Activation of Mitofusin2 by Smad2‐RIN1 complex during mitochondrial fusion. Mol Cell. 2016;62:520‐531. 27184078 10.1016/j.molcel.2016.04.010 PMC4877164

[55] Miller I , Min M , Yang C , et al. Ki67 is a graded rather than a binary marker of proliferation versus quiescence. Cell Rep. 2018;24:1105‐1112. 30067968 10.1016/j.celrep.2018.06.110 PMC6108547

[56] Yerushalmi R , Woods R , Ravdin PM , et al. Ki67 in breast cancer: prognostic and predictive potential. Lancet Oncol. 2010;11:174‐183. 20152769 10.1016/S1470-2045(09)70262-1

